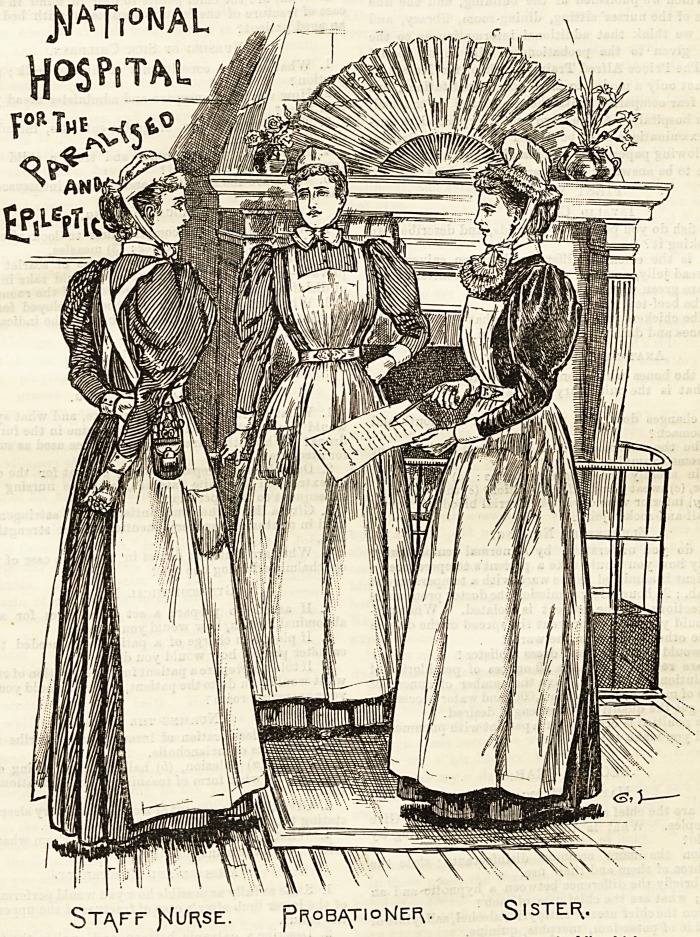# The Hospital Nursing Supplement

**Published:** 1896-03-28

**Authors:** 


### "The Hospital" Nursing Mirror.

Being the Extra Nursing Supplement of "The Hospital" Newspaper.

[Contributions for this Supplement should be addressed to the Editor, The Hospital, 428, Strand, London, W.C., and should have the word "Nursing" plainly written in left-hand top corner of the envelope.]

### News from the Nursing World.

### ROYAL VISIT TO LANCASTER.

Brilliant summer-like weather prevailed on Tuesday, when T.R.H. the Duke and Duchess of York visited Lancaster to open the new infirmary buildings, of which the foundation-stone was laid on the occasion of their marriage. This is the first Royal visit to the town since 1851, when the Queen was presented with the keys of the ancient castle, and it naturally aroused a considerable amount of enthusiasm. The guests were received by the Mayor (Alderman Huntington), whose daughter presented the Duchess with a shower bouquet of red and white roses. After the delivery of an address of welcome from the Corporation the infirmary was duly declared open by the Duke of York, who made a pleasant little speech, congratulating the people of Lancaster on the ample accommodation and modern improvements of the new hospital, and finally, amid much applause, announced that the Queen, at his request, had given her permission that for the future the new building should be known as the Royal Lancaster Infirmary.

### CHILDREN'S HOSPITAL, GREAT ORMOND STREET.

The Duke and Duchess of York visited the Hospital for Sick Children, Great Ormond Street, on Friday, March 20th, and were taken through the wards by the chairman, Mr. Arthur Lucas. Immense pleasure was given to the small patients by the pretty toys distributed by the Duchess.

### HONOUR WHERE HONOUR IS DUE.

Miss Selina Bland, matron of the Poplar Hospital for Accidents, was presented on Tuesday last, after the annual meeting at the hospital, by Dr. Edwin Freshfield, LL.D., Receiver-General and Knight of Justice of the Order of the Hospital of St. John of Jerusalem, with the badge of Serving Sister of the Order, to which honour she has been unanimously elected by the council and chapter with the approval of the Queen and H.R.H. the Prince of Wales. Miss Bland was appointed matron to the Poplar Hospital last May, and hearty recognition of her excellent management is recorded in the present annual report, the committee stating that "they believe the patients have never been better cared for than they are by her and her staff. She has in every detail maintained the high character she brought with her from the London Hospital, and does credit to that grand hospital where she received her training." Miss Bland's many friends in the nursing world, especially "old Londoners," by whom as "Sister Gloucester" she was so well known and esteemed, will rejoice at the well-deserved distinction conferred upon her. We offer her our sincere congratulations.

### WANTED—GOOD FEVER NURSES.

When will people realise that fever patients want good nurses? In spite of the many inducements offered to those willing to join isolation hospitals—in the matter of pay, holidays, pleasant quarters, and good food—the supply of suitable women remains extremely limited. Charge nurses who hold a certificate for general nursing take a position in a fever hospital equivalent to that of a ward sister. They have to supervise and instruct probationers, and they get, in their turn, much experience and knowledge of a very valuable branch of work. No private nurse can be considered thoroughly efficient who has not gone through a period of fever nursing. When the general hospital training is finished, many nurses are puzzled as to their best procedure, and make a grievance of having to leave because of the admittance of a fresh contingent of probationers to participate in the advanfages enjoyed by their predecessors. Yet the suggestion of the matron that there is a vacancy at one of the beautiful hospitals of the Metropolitan Asylums Board is received by the departing nurse with scant favour. It cannot be fear of personal infection, for that the nurse in a general hospital faces daily in the outpatient department, but it is rather the isolation from her friends and consequent dulness which is dreaded. Now that the principles of disinfection are understood, the fever nurse is unlikely to carry danger to those outside the walls, and the dulness is after all only comparative. There is plenty to do, and plenty to learn, and a library, tennis court, and piano make a nurse's life in a good isolation hospital much more agreeable than is imagined by those acquainted only with the outside of the walls.

### PREMATURE PROMOTION.

Among so many instances of indifference to the needs of sick paupers on the part of boards of guardians all over the country, it is cheering to find that the Bradford Board of Guardians have refused to sanction the appointment as charge nurse of the female wards of an insufficiently trained woman. The report of the Nursing Staff Committee, laid before the board the other day, stated that Nurse Lee had been promoted to this responsible position, and that following upon the appointment two nurses were reported for insubordination and required to resign their posts. One of the Guardians remarked that Nurse Lee was only a probationer in the workhouse, and had been placed over the heads of nurses of wider training and experience; he moved that the matter be referred back to the committee for further consideration. Nurse Lee not long since had complained to him of the insufficiency of the training she was receiving at the workhouse. The amendment was finally carried by the casting vote of the chairman.

### SMALL-POX AT GLOUCESTER.

Gloucester is paying a heavy price for the crass ignorance of its inhabitants, which for many years past has made that city an anti-vaccinationist centre.

Small-pox is raging furiously, despite gallant efforts on the part of the medical officers to arrest the progress of the epidemic. The District Nursing Association is working nobly, has engaged an entirely distinct staff to deal with the small-pox cases, and arranged for a separate house for their accommodation. The society has determined, "in strict alliance with the sanitary authority, to leave no stone unturned, and to put on any nursing strength necessary," and for this purpose is appealing to the public for funds to carry on the battle. A sub-committee, composed of Dr. Batten, Mr. H. E. Waddy, the Rev. A. C. Eyre, and Mr. George Whitcombe, has been constituted, and given absolute authority to take any necessary steps, and pecuniary help has been promised from several quarters already. A deputation from the association waited upon the Guardians the other day to ask for assistance, and a grant of £150 was unanimously voted, "without prejudice to further sums." That the resources of the association are taxed to the utmost may be gathered from the fact that at least a hundred cases are, it is said, being nursed in their own homes at the present moment. Surely Gloucester will at last learn wisdom by bitter experience, for this is, we believe, the third time in something like thirty or thirty-five years that the city has been attacked by this terrible and preventible scourge.

### A WISE PROPOSAL OVERRULED.

We are sorry to see that, in spite of the sensible advice lately given by Mr. Bircham, Poor Law Inspector for Wales, the St. Asaph Board of Guardians has rejected the proposal to grant a subscription to the Denbigh District Nurse Fund by sixteen votes to thirteen. It was stated by the Chairman that, of the 3,147 visits paid in 1895 by the nurse, 2,471 were to pauper patients, and several of the Guardians urged that the truest economy in the long run would be to support the work of trained nurses in the district; but, apparently, the immediate expenditure of the extravagant sum of one guinea was too alarming, and the Board has lost an opportunity of proving itself wise in its generation. It is a serious question whether any nursing association should undertake the nursing of outdoor paupers unless their expenses in so doing are repaid by the guardians, for all money so expended is but in relief of the rates, seeing that district nursing is now recognized by the Local Government Board as coming within the proper functions of the poor law authorities, and subscriptions to district associations as a proper way of undertaking the duty.

### A MEDICAL WOMAN AT ABERDEEN.

At a recent meeting of the directors of the Royal Infirmary, Aberdeen, Colonel Allardyce, LL.D., in the chair, an application came up for consideration from a gentleman who asked that his daughter, a student in medicine at Aberdeen University, might be permitted to attend the classes and practice of the infirmary. The request had been made at a former meeting, and referred to the Medical and Surgical Staff Committee, who now reported that "they saw no reason why the applicant should not be admitted to the ordinary practice of the hospital along with the other students." Permission was accordingly granted by the directors.

### BURY DISPENSARY HOSPITAL.

To the regret of all connected with this hospital, Mrs. Eilbeck has resigned the post of matron, which she has held for thirteen years. Very gratifying assurances of the high esteem which she has won for herself during these years of faithful service have come from all quarters, the Board of Management giving a cordial testimonial, and the Ladies' Committee having also presented her with an illuminated testimonial and a purse containing £51. From the nurses Mrs. Eilbeck received a silver tea-service, and the servants gave her a china tea-service, a copper kettle, and other small tokens of their regard. Miss Scott, of Bolton, has been appointed matron in succession to Mrs. Eilbeck.

### YORK HOME FOR NURSES.

This Home has done well during the past year, the fees earned by the nurses amounting to £3,066 18s. 6d., being an increase over those for 1894 of £404. Also, whereas the sum of £106 6s. 10d. was due to the treasurer last year, there is now a balance in hand of £104 11s. 10d. The staff numbers a sister in charge, three "assistant teachers" (what does this term mean, by the bye?), seventy nurses, and five district nurses. The nurses' work amongst the poor is evidently appreciated, for the working men of York contributed a considerable portion of the sum collected from house to house towards the "Sick Poor Fund," whereby they seem to have shown the way to the churches in York, for it is reported that an appeal for collections met with small response. An arrangement has been made with the officers of the garrison by which the services of a special district nurse have been secured to attend cases of illness among the families of the married soldiers.

### SHORT ITEMS.

The nurses of the Southampton branch of the Queen's Jubilee Institute sent some beautiful flowers to the funeral of Prince Henry of Battenberg, and Princess Beatrice, who is patroness of the institute, has written an autograph letter thanking them for the kindly token of sympathy.—The Johnstone and Elderslie District Nursing Association has lost a good friend by the death of Mrs. Finlayson, of Murchiston, who had been a hon. member of the committee since the association was started.—At the annual meeting of the Spalding branch of the Lincolnshire Nursing Association, it was stated that the past year had been financially satisfactory; 2,025 visits were paid to 72 patients.—The annual meeting of the Knottingley (Yorkshire) Nursing Association was recently held, and a satisfactory year's report presented. Nurse Hey, and Nurse Potts, her successor, attended 250 patients during the year, and paid altogether 3,600 visits.—It is stated that a question will shortly be asked by the Sir Walter Foster in the House of Commons with reference to pauper nursing in workhouses, with a view, we hope, of securing the appointment of universal trained nursing. — The Countess of Iddesleigh and Lady Elizabeth Northcote visited the wards of the Chelsea Hospital for Women this week. — The *Lady's Pictorial* for March 21st contains an account of the Pension Fund nurses' presentation to Princess Maud of their wedding gift and a drawing of the tea table.

### Private Nursing.

By Sister Grace.

### IV.—PRIVATE NURSES—SURGICAL CASES.

### Accidents.

This week we must consider surgical nursing and operative work as carried out in private houses. Under surgical cases we may place all kinds of accidents—the numerous fractures liable to occur to all, burns, though these are very rare, and diseases involving surgical dressings, but little or no operative treatment, such as various forms of hip disease, and scrofulous abscesses, with all their distressing complications, many cases of cancer not suitable for operation, and others too numerous to mention.

In nursing fractures in private houses, you will miss the convenient fracture-board of hospital use, and will have to exercise your ingenuity in utilizing all kinds of strange materials for that and other purposes. Except in cases of fractured thigh or knee, an absolutely flat bed is not essential, though highly desirable, therefore with fractures below the knee it is possible to arrange a flat board under the leg, where the splints are applied, such as a pastry board, or tea-tray turned upside down. Necessity will make you thankful for strange auxiliaries; but for fractures of the femur or patella, it is imperative that the whole bed should be level; a new straw palliasse will admit of this if the upper mattress is firm, and not too thick.

Serious accidents from the hunting-field, &c., you will often meet with, such as concussion, fractures of the skull and of the pelvis, and injuries to the spine; the two last should if possible be nursed on a water-bed, the almost immovable condition in which it is necessary to keep the patient renders the tendency to bed sores very great. If the circumstances are such as to make it impossible to stand the outlay necessary for buying a water-bed, it is now easy to hire one for a small sum.

In cases of serious injury to the spine, as you have doubtless had opportunities of observing in the wards, the frequent effect is partial or complete loss of sensation, and if the injury is sufficiently high up, sores will sometimes appear in twenty-four hours from the time of the accident. Naturally in these cases the time of suffering is not prolonged. Usually, however, the injury is lower down, and the patient may linger many weeks. The whole condition is very distressing and most painful to witness. Little can be done except to make the sufferer's last days as comfortable as possible.

With regard to bed sores generally, every nurse has her own favourite method of prevention and cure, so that I need not enlarge upon the subject, except to advise you not to be wedded to one particular form of treatment. Different skins require different applications; some are by nature so dry that the use of spirit is not advisable; some do not take kindly to ointments, and do better with spirit only; and others again do not answer to either method, and seem to progress well with frequent washing, plenty of soap (well rubbed in with the bare hand), and a little powder dusted on afterwards. Nurses consider that bed sores are a disgrace to them, and as a rule they would be so, but sometimes it is absolutely impossible to prevent them, especially when the patient has been allowed to get into a neglected condition before you are brought to the case. Do not let any feeling of timidity, or thought of its existence being a reflection on your powers as a nurse, prevent your mentioning the first appearance of such a thing to the doctor.

### Operative Work.

This work in private houses certainly presents many difficulties to the nurse, unknown to her co-workers in hospitals. There we have everything arranged with the utmost exactitude for the management of each case, and all our appliances are of the best form, size, and shape, adapted to modern surgery. Far different is the case of the private nurse; from first to last it is a matter of "making things do," and I have often been lost in admiration at the pitch of perfection to which some clever nurses have brought the art. As every case is different in its surroundings, I cannot help you much with suggestions, and can only say that it is work that greatly taxes a woman's ingenuity.

Usually the surgeon brings his own dressings and drugs, so that you have not that to think of; but you must collect as many basins and bowls as you think you are likely to want; and as there is no convenient theatre sink at hand, a couple of pails, a large quantity of hot and cold water, and plenty of towels will also be wanted. The table on which the operation is to be performed is another difficulty, and is generally not thought of till the nurse arrives—perhaps only the night before the operation.

The kitchen table usually answers best, I think, because it is firm and square on its legs, and of a convenient height and width. You must be prepared to encounter many difficulties in operative work in cottages. I remember being sent for at eleven o'clock at night to help a doctor in an outlying cottage on a moor, and a terrible experience we found it. No basins, no water, no table, no lights, no towels, no anything. After diligent search, assisted by an ancient dame who "did for" the patient, I found a treasure in the shape of two tallow candles, but no candlestick. I put them in bottles, and with the addition of two dingy pewter spoons, polished hurriedly on the hem of my skirt to a meretricious splendour, and fixed to the necks of the bottles with strapping, so that the bowls made reflectors to the feeble flames, we had quite an illumination. I greatly admire the cleverness with which some private nurses meet and overcome all sorts of difficulties, and I am sure they must experience real pleasure in successfully surmounting obstacles calculated to make the boldest despair, but I imagine it is a pleasure of which a little will go a long way. I have always thought and said that it is comparatively little credit to us in hospitals to have everything go smoothly, whereas in even the best ordered private houses the nurses must always encounter difficulties and trials of which we know nothing. All honour to them when they cope with them uncomplainingly and successfully.

### Burdett's Official Nursing Directory.

We are asked by the Editor of "Burdett's Official Nursing Directory" to explain to all those who are interested in this publication that the initial labour attendant upon the verification of particulars has made its speedy production impossible. The editors have spared no pains to render the publication truly valuable and accurate, and to justify its official character. They also ask the co-operation of hospital authorities, to whom in a short time they will appeal, asking them to check all particulars relating to the training and employment of nurses stated to be under their control. The editors take this opportunity of thanking the many matrons, medical superintendents, and others who have already rendered them valuable aid in the heavy task of bringing together a mass of hitherto unrecorded facts relating to the education, employment, and status of nurses. We feel sure that the appreciation of the medical profession, nurses, and the public generally of a work which will supply a serious want in the nursing world, will ultimately repay the editors for their arduous undertaking.

### On Certain Aspects of the Nursing Question as Seen in England and Germany.

By a Certificated Midwife.

### IV.—THE HEBAMMEN SCHULE.

The "Vorstand," or, as we should say, "Head" of this School, was a man of great reputation. He was good enough to admit me as a pupil, but he could not give me board and lodging, as every bed was occupied, and the term had already commenced before I came out of Ludwig's Spital. As far as I remember there were about forty scholars. They were chiefly of the working-class, and were dressed in the usual costume of German peasant women—a skirt of one stuff, and a short jacket of another, a large checked apron, and with a small kerchief tied over neatly-braided hair. The few exceptions were women of a rather better class, who wore clothes which called for no remark, being always neat and nothing more. There was no attempt at a uniform, which always entails expense. There were three terms in the year, beginning on January 1st, May 1st, and September 1st. At the end of three months the examination took place, which, I think, occupied about a week. Then there was a distribution of the certificates, a final lecture from the Vorstand, and addresses from both the Protestant minister and the Catholic priest on the duties and responsibilities of the profession these pupils were about to take up. Especially they were instructed as to the baptism of the infants, which they were expected to arrange for ere they gave up the charge of any patient, or to perform themselves in a decent and reverent manner in case of a babe being in immediate peril of death, and no minister at hand. Leave-takings over, a fortnight's vacation was the rule before the next set of learners arrived. This gave time for a thorough cleaning of the hospital, and the care of the patients was left to the residents and two or three of the pupils who were considered to want a little more training in practical details.

The routine of our daily instruction during term was as follows. There was a large lecture hall, which was called the theatre, and in this we assembled three times a day. The first lecture was always given by the "Herr Doctor" himself. Punctually at eight a.m. his quick steps were heard, and after he had made the round of the wards, where he found all the pupils who had patients under their charge waiting to give their report and receive orders (always, however, given through the resident midwife, and not direct to the pupils), he came into this hall, followed by his train of learners. We sat in a semicircle, each with our "lehrbuch" or manual in hand, out of which we read in rotation, every woman the same paragraph. This theatre was furnished with a large number of diagrams hung on the walls, a sufficiency of leathern dummies, and a female skeleton, besides a variety of separate bones. It was on the skeleton that the pupils took their first lesson, and until they knew the framework of the human body in all its divisions and most of its details they were not taught anything about its organs or functions; and I think about three weeks elapsed before we got on to the subject of childbirth. I shall say more about the manual later. Now, I want to point out that, though it was very simple in its language and clear in method, the teacher took nothing for granted, but explained it and every word of our day's lesson as we went on, making certain of every step ere he allowed us to take another. Questioning, illustrating, scolding by turns, his rapid eye gauging the ability and the attention of each, this learned professor bent his whole mind to the task he had undertaken, and this hour was most carefully prepared for by all the women, though it was not the most dreaded. When it was over we took our lunch (we had breakfasted at six, remember), and then assembled for a second time in the theatre, where we repeated all we had learned to the housemaster, an important member of the staff, whom I will describe later. Then we returned to our ward duties. Dinner was at noon, and in the afternoon the house surgeon gave us some instruction of a varied character, also in the theatre. Next came our modest cups of coffee, then the resident midwives held their classes of practical teaching in one of the large wards, and, finally, after bathing our infants and attending to their mothers, we awaited the coming of the "Herr Doctor," standing by our patients. This was the worst hour of the twenty-four. In he came, followed by the house surgeon and both the residents; and without asking a single question waited for the pupil in charge to relate the history of each new case that had been put to bed since last evening. Without hesitation or circumlocution the pupil was expected to give an accurate and succinct account of the mother—her age, status, date of entry, health during her time of expectation, if *primipara* or no, the whole course of labour, with the time of birth, the sex, weight, condition of the infant, winding up with the temperature, pulse, and general condition of the mother at that moment. This was an ordeal in truth. Many were the rebukes and caustic remarks which fell from the lips of the learned and much-to-be-feared chief at this hour; but it was excellent practice for us pupils. We learned to be concise and absolutely accurate, as well as to pick out the salient points of each case and to reject all irrelevant details. There might be three or four new cases—there might be only one; but I do not think I ever recollect a day on which no birth at all took place. For in giving an account of our daily routine I have perforce left out all mention of the labour ward duties which called some of us away from almost every class or lecture, except that of the "Vorstand" himself, which we were never allowed to miss, unless under very unusual circumstances. But, whether there were many or few new cases, the conscientious doctor never missed going to every bedside, even of the convalescent, and seeing for himself the state of every inmate; and woe betide that nurse who was unready with her report!

"Third day, temperature normal, all goes well," with certain technical details, was the sort of thing he expected to hear. But his eye was vigilant to a degree, and he detected the slightest deviation from fact, or want of observation, and then his ire rose. By half-past six he was generally out of the house, and then we all breathed freely and could sit down to our evening meal in peace. As I lodged out of the house, I left soon after seven o'clock, to return at the same hour next morning, unless I were wanted for the night.

### Royal British Nurses' Association.

### SESSIONAL LECTURE.

"The Village of Palaces" was the title of the excellent lecture on Old Chelsea given by Miss de Pledge on March 20th at the offices of this association. Dr. Bezley Thorne presided, Miss Thorold, Mrs. Dacre Craven, and a number of nurses in uniform being also present. The lecturer appeared to possess a thorough acquaintance with the annals of a most interesting district, and she conveyed much information in a particularly attractive form to her attentive audience. It was suggested that Miss de Pledge should be asked at some future time to give another lecture dealing with a later period of the history of Chelsea which time had not permitted of her touching on Friday evening. A pleasant discussion followed the address, a cordial vote of thanks being offered to the lecturer, and another to Dr. Bezley Thorne for his kindness in taking the chair.

### Dress and Uniforms.

By a Matron and Superintendent of Nurses.

### NATIONAL HOSPITAL FOR THE PARALYSED AND EPILEPTIC, QUEEN'S SQUARE, BLOOMSBURY.

The uniform worn by the staff of the National Hospital is as quaint and as pretty as the old-fashioned square in which the building is situated. Navy blue serge is the material of which the sisters' dress is composed, and it looks both neat and serviceable. The bodice is quite plain, buttoned down the front, and the skirt just touches the ground all round. A linen apron with large square bib amply covers the dress, and plain linen cuffs and collars give a finish at the neck and wrists. The cap is very picturesque, resembling in style those worn by the Normandy peasants, and is most becoming to the wearer. The high-peaked crown is gathered into a band which fits round the head, and is edged with two narrow rows of gophered lace frilling. Large muslin strings bordered with lace tie under the chin and complete this charming head-dress. The staff nurses' dress is narrow blue and white striped galatea made quite plain, with rather full sleeves. The linen apron has bands attached to the bib which cross at the back. They wear the pretty "Sister Dora" cap, always so attractive, which ties with narrow strings in a neat little bow in front. Pink and white striped galatea is worn by the probationers, and is the only point of distinction in costume between them and the staff nurses.

STAFF NURSE. PROBATIONER. SISTER.

### Training in New South Wales.

### PRINCE ALFRED HOSPITAL, SYDNEY.

Accounts have appeared in The Hospital from time to time showing the excellent system of training which has been introduced with marked success at the Prince Alfred Hospital, Sydney. The beautiful building, with its charming outlook, is familiar to everyone who knows Sydney, and the Nurses' home is well worthy of the institution whose nursing and domestic staff are housed there. Our readers will remember the plans which we published of the building, and the fine proportions of the nurses' sitting, dining-room, library, and study, and we think that additional information as to the instruction given to the probationers will be of special interest. The Prince Alfred Training School for Nurses at Sydney is not only a model to all Colonial institutions, but it need not fear comparison with those attached to the leading English hospitals. As we have before explained, a succession of examinations must be passed by the probationers, and the following papers give an idea of the questions which which have to be answered on the several occasions:—

### FIRST YEAR.

#### Invalid Cookery.

1. What fish do you prefer for invalids, and describe two ways of cooking it?
2. What is the essential difference between calves' feet jelly and bread jelly, and how do you make the latter?
4. How are green vegetables cooked?
5. Describe beef-tea, raw and cooked?
6. Describe chicken panada, and say what you would do with the bones and dark flesh of the chicken.

#### Anatomy and Physiology.

1. Name the bones which constitute the cranium.
2. (*a*) What is the pulmonary circulation? (*b*) What are its uses?
3. What changes does food undergo (*a*) in the mouth and (*b*) in the stomach?
4. Describe the nature of reflex action and explain your answer by some examples.
5. Explain shortly the following terms: (*a*) Pelvis, (*b*) mitral valve, (*c*) sweat gland, (*d*) respiration, (*e*) thoracic duct, (*f*) urea, (*g*) inferior vena cava, (*h*) arterial blood, (*i*) portal vein, (*j*) ball-and-socket joint.

#### Elementary Nursing.

1. What do you understand by a normal temperature? State briefly how you would take a patient's temperature.
2. A patient is admitted to the ward with a temperature at 103 deg. Fah.; 24 hours after admission the doctor pronounces the case infectious and the patient is isolated. What precautions would you take to prevent the spread of the disease amongst the other patients in the ward?
3. How would you apply and dress a blister?
4. You are required to make 20 ounces of perchloride of mercury solution 1 in 4,000. Give the number of ounces of perchloride of mercury lotion (1 in 1,000) and water necessary, in order to get the quantity and strength desired.
5. An ice poultice is ordered for a patient with pneumonia. How would you make it?

### SECOND YEAR.

#### Materia Medica.

1. What are the chief symptoms of irritant poisons? Give three examples. What is the treatment of poisoning by carbolic acid?
2. Mention the more common disinfectants; state the action of three of them and their use.
3. State briefly the difference between a hypnotic and an anæsthetic; what are the chief uses of each?
4. Mention the chief uses of antipyrin, alcohol, salicylate of soda, bromide of potassium, morphia, quinine.

#### Surgical Nursing.

1. (*a*) State the different methods employed to stop bleeding from a wound on the surface of the body. (*b*) What would you especially attend to in the nursing of a case of concealed hæmorrhage after an injury to the chest?
2. Explain the following terms: (*a*) Traumatic fever; (*b*) inflammatory fever; (*c*) septic traumatic fever; (*d*) septicæmia; (*e*) pyæmia.
3. State the four conditions necessary in order that microbes or disease germs may grow and thrive. What is an antiseptic? Name the principal antiseptics used in the treatment of wounds, stating which are poisonous when taken internally.
4. State in detail how you would prepare a patient for the operation of amputation of the foot.
5. What are the chief points to bear in mind in nursing a case of fracture of the femur complicated with bed sores in an aged patient?

#### Nursing of Sick Children.

1. What are the constituents of human milk; give proportion?
2. How would you prepare and administer bread jelly and beef juice?
3. Tell all you know of gastro-enteritis, including the dietetic treatment.
4. How would you recognise and treat a child suffering from prolapse of the bowel?
5. What are the symptoms and signs of intussusception of the bowel?

#### Medical Nursing.

1. What are the chief complications to look after in—(1) typhoid fever; (2) scarlet fever; (3) measles.
2. You are engaged to nurse a case of scarlet fever in private—state what precautions you would take in the way of disinfection, and in the arrangements of the room.
3. What are the common methods employed for the reduction of high temperatures? What are the indications for the use of stimulants?

### THIRD YEAR.

#### Ophthalmic Nursing.

1. What precautions would you take, and what symptoms would you watch for when using atropine in the form of eye drops? What other drugs are sometimes used as substitutes for atropine?
2. Describe the preparation of a patient for the operation of extraction of senile cataract, and the nursing required subsequent to the operation.
3. Give a list of the usual antiseptic and astringent lotions used in ophthalmic surgery, mentioning the strength in each case.
4. What are a nurse's duties in tending a case of purulent ophthalmia affecting one eye?

#### Gynæcological Nursing.

1. If asked to prepare a set of sponges for a case of abdominal section, how would you proceed?
2. If placed in charge of a patient who needed to have a catheter passed, how would you do it?
4. If told to prepare a patient for the operation of curetting, what would you do to the patient, and how would you prepare your operating room?

#### Nursing the Insane.

1. Give a classification of insanity and describe the chief characteristics of melancholia.
2. Define (*a*) delusion, (*b*) hallucination, giving examples of each. In what form of insanity are hallucinations usually seen?
3. Give the management of a case of ordinary sleeplessness, stating the common causes of this condition.
4. What is meant by somnambulism? From what does it arise? Give the management of a case.

#### Massage and Electricity.

1. State as fully as possible how you would perform massage of the lower limb after a case of fracture of the upper third of the femur.
2. Describe a galvanic battery, and explain the following terms: Constant current, interrupted current, anode, cathode, electrode, rheophores.
3. Enumerate the different procedures usually adopted in the performance of massage, and state what is meant by the terms centripetal and centrifugal.
4. In what diseases are the forms of electricity most often used, and in what are the results most beneficial?

### A Book and its Story.

"THE DAYS OF AULD LANG SYNE."

The Rev. W. Watson, who writes under the *nom de plume* of "Ian Maclaren" was, for some years, a minister in the parish of Logiealmond, in Perthshire, and every page in this volume of short stories is steeped in the local colouring of the Highland hills. It is from the southern slopes of the breezy Grampians that the author has drawn his inspiration. For mile after mile the great purple mountains stretch, with the dark brown waters of the Almond at their feet, roaring in some places over boulders, and in others flowing placidly along beneath the overhanging boughs of pine and birch. Far away to east the eye can almost reach the German Ocean; and in front the wide plain of Strathearn displays its wealth of cultivated fields, plantations, and pasture lands; the Castle Rock of Stirling is just visible on the southern horizon; while to the west the view is bound by the distant ranges which bend their stern brows above "lone Glenartney's hazel shade." The district is rich in historical associations. Glenalmond is traversed by the road along which Prince Charlie marched during the disastrous retreat which ended at Culloden. There are the remains of a Roman camp; and to remind the antiquarian of an equally remote period, the spot to which tradition has assigned the grave of Ossian is pointed out. The village in which "Ian Maclaren" was at one time a well-known figure lies at the eastern extremity of Glenalmond, close to Trinity College, one of the great public schools of Scotland, with its towers, its cloisters, its chapel, and its spacious playing fields. All around are shooting lodges and grouse moors. But in the fascinating volume before us we are not brought in contact with the boys of Trinity College, or with the tenants of the shooting lodges. The author has preferred to take us among the farmers and villagers of the glen, for one cannot doubt that while writing "The Days of Auld Lang Syne" Ian Maclaren was thinking of his highland parish, and that many of the characters which he describes are drawn from living people.

Of the ten stories of which the volume consists, the longest, "For Conscience Sake," is perhaps the best. John Baxter, the farmer of Burnbrae, is a tenant on the estate of Lord Kilspindie. A new factor has just been appointed. "He's a cousin of an English lord," one of the characters explains, "whose forbears got a title by rouping their votes, an' ony conscience they hed, tae the highest bidder in the bad auld days o' the Georges —that's the kind o' bluid that's in his veins, an' it no clean. His fouk started him in the airmy, but he had tae leave—cairds or drink, or baith. He was a wine merchant for a whillie an' failed, and then he was agent for a manure company, till they sent him aboot his business." This gentleman, having made England too hot to hold him, has now the power almost of life and death over four parishes, while his employer is travelling in the East; and he has resolved to eject the tenant of Burnbrae, whose lease is about to expire, on the ground that he is a Dissenter. Knowing nothing of Scotland, the new factor is astonished when the minister of the Established Church remonstrates with him. "Who gave you authority," the minister asks, "to interfere with any man's religion? You know neither the thing you are doing nor the men with whom you have to do. Our farmers, thank God, are not ignorant serfs who know nothing and cannot call their souls their own, but men who have learned to think for themselves, and fear no one save Almighty God."

But the factor will listen to no appeal; and the farmer is informed that he must choose between attending the Established Church and leaving the place in which he and his forefathers have lived for generations—between his farm and his conscience. He and his wife Jean are now old people, and the pain of leaving the old home is almost more than they can bear.

The story of their struggle against the temptation to yield, and of the triumph of principle with which the struggle ended, is full of touching pathos, and, at the same time, free from morbid sentiment. The two leave their farm; but there is an appeal unto Cæsar in the shape of Lord Kilspindie, who reverses the decision of his factor, and everything ends happily.

Then there is the story of the village cynic, one James Soutar, who had a "nippy tongue," and whose gift of speech was aided by his eyes. "They were blue—not the soft azure of the South, but the steely colour of a Scottish loch in sunshine, with a north-east wind blowing—keen, merciless, penetrating blue. It gave a shock to find them fastened on one when he did not know Jamie was paying any attention, and they sobered him in an instant. Fallacies, cant, false sentiment, and every form of unreality shrivelled up before that gaze. . . . He had a way of watching an eloquent stranger till the man's sentences fell to pieces and died away in murmurs before he said 'Ay, ay' that was very effective." Yet, cynic as he was, Jamie was a favourite in the glen, and, when he lay on his deathbed, one of those who came to see him expressed some wonder that he had never been married.

"The Glen," he answered, "thocht me an auld cankered bachelor, an' a've seen a lass leave her lad's side on sicht of me. Little they kent." And then he tells the story of how, in his youth, he had met a girl whom he loved: a story of exquisite beauty and simple pathos.

"We cudna be mairrit till the summer, an' we agreed tae write nae letters tae set the fouks' tongues gaein'; we wanted tae has oor ain secret. So we trysted tae meet aince a week at a stile in the woods atween here and Kildrummie, an' we hed seeven evenin's thegither; that's a' we ever saw o' ane anither in this warld."

On the eighth evening he went to the place of meeting, but "Menie" did not come; and in a short time he heard she was dead. That was the secret of his life; and now his own last hours have come. Two women who are nursing him think that all is over. "He hes the look, an' his hands are as cold as ice; feel his feet, wumman," said Kirsty. "A' canna find them," said Elspeth, making timid explorations.

"They used tae be on the end o' ma legs," remarked the dying man, as if uncertain where they might now be placed.

Elspeth started back and looked at him, but his eyes were closed, and he gave no other sign of consciousness.

"A'll no meddle wi' him again," said Elspeth solemnly, "though a' set here for a week. He's a queer body; he gaed his ain wy a' his life, an' tak ma word for't, Kirsty, he'll hae his own wy o' deein."

When the first ray shot through the window and trembled on the bed, the man raised himself and listened. He shaded his eyes with his hand as if he were watching for someone and could not see clearly for excess of light. "Menie," he cried suddenly, in a new voice, "a've keepit oor tryst."

In these pages the Scottish peasant stands before us, his character, partly, perhaps, the result of more than three centuries of such a system of education as we in England have known for only one generation, but still more, perhaps, of the constant struggle to overcome difficulties, of the tradition of independence handed down from father to son, and of a certain enthusiasm peculiar to the race. Ian Maclaren's characters in "The Days of Auld Lang Syne" are living people to his readers, described by a gifted and fascinating writer.

* "The Days of Auld Lang Syne." Ian Maclaren. (London: Hodder and Stoughton. 1895.)

### The Misuse of a Nurse's Uniform.

An esteemed correspondent writes to us as follows: Knowing that you take an interest in all matters pertaining to the nursing profession, I feel sure you would be glad to know of a new field for their energies and to what remarkable uses their uniforms may be put. Ever anxious to increase my slender earnings as a working woman, I saw and answered in person the following advertisement in the *Daily Telegraph* of March 17th:

WOMAN (Young), tall, good appearance, wanted to distribute circulars to ladies in the street. Wear nurse's uniform. No fringe. Must be self-confident. Call after eleven. Madame, 272, Gloucester Terrace, Bayswater.

Being almost conceited enough to suppose I fulfilled the necessary qualifications for the post, I mentally contemplated brushing back a distinctly refractory fringe, and with such resolutions I summoned up enough courage to ring the bell at the door of Madame Maude Trautner, dressmaker, 272, Gloucester Terrace, Bayswater, and explained the purpose of my visit by presenting the above advertisement. The door was opened by a man who received me with that class of reassuring smile which is usually reserved for probable customers, but on explaining my errand I was rather abruptly handed over to a person called "Maude," who I imagined was "Madame" herself.

The lady received me in the narrow hall, and, after carefully taking my measure, remarked that "I don't think you will quite suit me." Here was a slight to my vanity, but, being a persistent young woman, I explained I should like to know exactly the nature of the services she would require, assuring her at the same time of my business capacities. I learnt that "the young lady of good appearance" would be required to distribute circulars, in the form of single leaflets, which enlarged on the merits of Madame Trautner's dress-making capabilities. Every well-dressed woman who passed along the streets between the hours of ten a.m. and six p.m. was to be presented with one of these advertisements. "But why in nurse's uniform? Is that a particular branch of your work?" I asked. "No, I have nothing to do with nurses; but I think the uniform most attractive, and would constitute a better medium of advertisement than the boy I usually employ for the purpose." "But may you adopt the uniform of any hospital indiscriminately?" I inquired. "Certainly; there is no objection that I know of." I then questioned "Madame" on the subject of her terms, as to whether there was a salary attaching to the post, to which she replied: "No, the chief payment will be a commission of 1s. on every order that could be traced to the circular." To my own mind this did not seem very liberal payment, so I put it to her again. "But is there absolutely no other salary besides this commission?" After a little hesitation, "Madame" thought she might "give a small salary" to the extent of 10s. a week, but with this I was to find my own uniform and distribute the leaflets for the hours already mentioned for the present, but, as the days grew longer, until sunset. I then asked her whether she had any fancy for the costume of any particular hospital, to which she replied she was indifferent on that point, provided it was the orthodox nurse's dress. The prospect of 10s. a week and possible, but not probable, commission of an occasional shilling, rather damped my ardour, notwithstanding the further attraction of disporting myself in that most becoming of costumes.

As I neared the door "Madame" grew quite affable, and added, "I think I am suited, but still, I should be pleased for you to call again in a day or two." I thanked her, and replied I would consider the matter; and as I went musing on my way through the busy thoroughfare of Westbourne Grove, I came to the conclusion that truly woman's work was varied, but hardly as yet valued at a high premium.

### Everybody's Opinion.

[Correspondence on all subjects is invited, but we cannot in any way be responsible for the opinions expressed by our correspondents. No communications can be entertained if the name and address of the correspondent is not given, or unless one side of the paper only be written on.]

### PRIVATE NURSING BY THE DAY OR HOUR.

"Trained Nurse" writes: I want very much to know if there would be any opening in London for a private nurse undertaking daily and hourly visits to cases where there is no sleeping accommodation for a nurse by the week, and where possibly her services are not needed all day and every day, but only at stated times. It seems to me that there must be many families in which there are cases of chronic illness to whom the expense of a private nurse at two guineas a week would be prohibitive, and to whom also the constant presence of an additional person in a small house where space is limited would be impracticable, who would joyfully hail the possibility of obtaining the services of a thoroughly-trained and competent nurse, say, for an hour or two each day, to render the numberless attentions which can only properly be done by a nurse. I fancy that an attempt to supply what is certainly a want among a section of the public should meet with success, and I should like to hear if this has been done at all in London, and what have been the experiences of nurses working on these lines?

### Minor Appointments.

Central London Sick Asylum.—Nurse Margaret Chick has been appointed Charge Day Nurse at this institution. She was trained at the Stockton-on-Tees Hospital, and has since held the appointments of assistant day nurse and charge night nurse at the Fulham Infirmary.

Eastern Fever Hospital, Homerton.—Miss Agnes Carson has been appointed Charge Nurse at this hospital. She received three years' training at the Kensington Infirmary, and has since been charge nurse at Mill Road Infirmary, Liverpool, sister at Kensington Infirmary, and superintendent nurse at Stroud Union, Gloucester.

Holy Cross Society of Trained Nurses.—Miss Margaret A. Ritchie has been appointed Sister-in-charge of St. Mary's Home, Filmer Road, Fulham, the maternity branch of the Holy Cross Society. Miss Ritchie was trained at the Radcliffe Infirmary, Oxford, afterwards taking up private nursing in the same city. Her last appointment has been in the capacity of sister over the maternity wards at the Kensington Infirmary. We cordially wish her all success.

Indian Nursing Service.—Miss Ethel Sykes has been appointed Nursing Sister in the Indian Nursing Service. Miss Sykes trained for three years at the Royal Free Hospital, and was afterwards charge nurse at the Essex and Colchester Hospital. She has our best wishes for health and success in her work abroad.

Stroud Union.—Miss Elizabeth Allen has been appointed Charge Nurse at the Stroud Union Workhouse Infirmary. She received her training at the Stafford General Infirmary, and has also worked at the Oldham Workhouse Infirmary, the Borough Hospital, Sheffield, and at the Havant Workhouse Infirmary.

Stockport Union Infirmary.—Miss H. Hudson has been appointed Charge Nurse at this institution. She was trained at Stafford General Infirmary and at Bristol Workhouse Infirmary, having since held the post of extra charge nurse and matron (*pro tem.*) of the Stockport Isolation Hospital, and nurse at the Keynsham Union Infirmary.

### Wants and Workers.

Wanted, to find a home or institution where an aged paralysed woman could be received permanently, either free or on very small payment, in or near London.—Y.

### News from Melbourne.

LADY BRASSEY, wife of the lately-appointed Governor of Victoria, has announced her intention of establishing over here a society for the prevention of cruelty to children on the model of that in England, in which she has long taken an interest. The Chief Commissioner of Police is aiding her in its formation.

Miss F. Farquharson has been appointed matron of the Melbourne Hospital. Miss Farquharson was trained in the Crumpsall Infirmary, Manchester, and was for some time matron of the Coast Hospital, Sydney, N.S.W. She was also for several years matron of the Alfred Hospital, Melbourne.

A sad death occurred among the nursing staff of the Melbourne Hospital last autumn. Nurse Emily Mary Fisher was an Englishwoman by birth, and was only thirty-one years of age. She was in charge of the septic and contagious diseases ward, and was considered to be one of the most efficient nurses in the hospital. She had sustained a very slight scratch on the hand, but took no notice of it, and continued at her post for some days, although not feeling well. When at last she had to give in she was already hopelessly ill.

The subject of snake bites is as interesting to Australians as to Indians, for Australia possesses two very deadly varieties of the reptile, the tiger-snake and the death-adder, though our proportion of venomous snakes is much less than in India, especially in the south. Still, every year there are many deaths from snake-bite, many of them resulting fatally because no one at hand has a notion what remedy to apply in the emergency. A few medical men, especially Dr. Halford, of the Melbourne University, have been studying the subject for the last thirty years, though nothing very definite has been arrived at. Lately Dr. Ralph, of the Sydney University, has obtained some successful results, and put them forth in a published form early in this year. Dr. Martin has also been engaged in researches on the subject, and quite recently (February) a self-styled "professor" has been making displays of an antidote in Melbourne, incidentally showing that ordinarily thick woollen stockings and mittens are sufficient protection against the bite of Australian snakes.

### Appointments.

CORK STREET FEVER HOSPITAL, DUBLIN.—Miss Carson Roe has been elected to the post of Assistant Superintendent and Matron at this institution. She trained at St. Thomas's Hospital, and has since held the appointment of night superintendent at the St. Marylebone Infirmary. We wish Miss Roe every success in her new work.

ROYAL WEST OF ENGLAND SANATORIUM, WESTON-SUPER-MARE.—Miss Edith Mawe has been appointed Lady Superintendent of this institution. We regret that her name was erroneously given last week.

### Where to Go.

ST. THOMAS'S HOSPITAL.—A lecture in aid of St. Thomas's Appeal Fund will be given in the Governors' Hall at the Hospital on Tuesday, March 31st, at nine o'clock p.m., on the "New Röntgen Photography," with limelight illustrations and practical demonstrations, by Mr. A. Campbell Swinton. Tickets, 10s. 6d. each, can be obtained by application to the Treasurer, the Counting House, St. Thomas's Hospital, S.E.

### Novelties for Nurses.

### SURGICAL ELASTIC HOSIERY.

MR. VINCENT WOOD has purchased the business of the Surgical Hosiery Company, Nottingham, and has established a "Surgical Elastic Hosiery" manufactory in London. He also issues a list of his goods, with special terms to doctors and nurses.

### Practical Points.

*Phthisis.—Do you consider that a nurse, in whose family there is a consumptive tendency, though herself strong and healthy, runs any risk of developing the complaint by nursing consumptive patient ?—S. A.*

The answer to this question will vary somewhat according as it is looked at from the theoretical or the practical standpoint. Theoretically, the constant mixing with consumptive folk ought to, and must, increase the chances of being attacked by tuberculosis. Practically, the experience of hospitals devoted to the reception of such patients is that their nurses do not suffer. Much, however, must depend upon surroundings. Anyone who visits the wards and corridors of the Brompton Hospital must admit at once how superior are the surroundings there to those which too commonly exist in the homes whence consumptives come; and although we say, without reserve, that a person nursing in a well-managed consumption hospital runs no more risk of tuberculosis than in many other kinds of work, and far less than in some, this argument does not apply to nursing patients in their own homes, amid the surroundings in which the disease has developed in the patient. We have but little doubt that the home nursing of a consumptive does involve an appreciable risk, which, however, may be reduced to extremely small dimensions if money be plentiful and if constant care be exercised. The less the wealth, and thus the power of taking precautions, and the less the willingness of the patient to submit to all sorts of regulations and restrictions, the greater will be the danger not only to the nurse but to other members of the family.

### Notes and Queries.

The contents of the Editor's Letter-box have now reached such unwieldy proportions that it has become necessary to establish a hard and fast rule regarding Answers to Correspondents. In future, all questions requiring replies will continue to be answered in this column without any fee. If an answer is required by letter, a fee of half-a-crown must be enclosed with the note containing the enquiry. We are always pleased to help our numerous correspondents to the fullest extent, and we can trust them to sympathise in the overwhelming amount of writing which makes the new rules a necessity. Every communication must be accompanied by the writer's name and address, otherwise it will receive no attention.

### Queries.

*E. K. M. E.* sends no name or address. Anonymous queries cannot be replied to.

(195) *Training.*—Can you tell me of any hospital where probationers are received at the age of 20, and will you also tell me if all nurses are required to be above a certain height ?—*E. M. H. and M. H. P.*

(196) *Nurses' Holiday Home.*—I want the address of the Nurses' Holiday Home at Hastings if you will kindly give it to me.—*Nurse R.*

(197) *South Africa or India.*—I am anxious to nurse in South Africa or India. Can you advise me where to apply for information ? I am fully certificated, and would take either hospital or private nursing.—*Anxious One.*

(198) *Hospital Nursing.*—Will you tell me at what children's hospital I can enter as non-paying probationer at the age of 18 ?—*Ethel.*

(199) *Nursing by the Day or Hour.*—Will you tell me how I should set about getting daily or hourly nursing in London ? I have been doing private nursing for some years, but find I cannot undertake any more night duty on account of health.—*A Constant Reader.*

### Answers.

(195) *Training (E. M. H. and M. H. P.)*—(Get "How to Become a Nurse," and then apply direct to the matrons of the various children's hospitals which you will find there given where probationers are admitted as young as 20. In making application state your height. Rules on that score are made in some institutions.

(196) *Nurses' Holiday Home (Nurse R.)*—We have made inquiries, and cannot find that there is any such home at Hastings.

(197) *South Africa or India (Anxious One).*—You will be very unwise if you go abroad with a view of taking up nursing without a definite engagement. Look in "Burdett's Hospital Annual" for the names of hospitals in South Africa, and write direct to the matrons. With regard to India, you can obtain a form of application for the Indian Nursing Service at the India Office, St. James's Park, or you might apply to the hon. secretary of the Up-Country European Nursing Association, the Hon. Mrs Neville Lyttelton, 21, Carlton House Terrace.

(198) *Hospital Nursing (Ethel).*—Get "How to Become a Nurse" (Scientific Press, 428. Strand, W.C.), and apply to the various hospitals there mentioned. We fancy few children's hospitals take probationers under 19 or 20 years of age.

(199) *Nursing by the Day or Hour (A Constant Reader).*—We publish to-day a letter on this subject in "Everybody's Opinion," which will no doubt elicit information from those who have tried nursing on this plan. We think there is a distinct demand for something of the kind, but you would need to be sure of a certain number of cases before starting on your own account. Consult the medical men under whom you have worked as private nurse.

### The Book World for Women and Nurses.

[We invite Correspondence, Criticism, Enquiries, and Notes on Books likely to interest Women and Nurses. Address, Editor, The Hospital (Nurses' Book World), 428, Strand, W.C.]

Baby Buds. By Ellis Ethelmer. (Mrs. Wolstenholme Elmy, Buxton House, Congleton. 1895. Price 1s.)

This is a little book which will appeal to people very differently according to the point of view from which it is regarded. It is an attempt to describe to the childish mind some of the main outlines of the principles of generation, or the means by which the propagation of the human race, as well as of other species, is effected. In its way the task is well performed, and we do not know that so difficult a subject could be more delicately dealt with. The main point —regarding which such a book will be sure to meet with criticism—is, as to whether it is worth while to deal at all with such a topic, especially if the information which is allowable is to be given in such an infantile form. The worst of all such books is their suggestiveness. Propriety forbids an out and out description in plain words, and therefore processes have to be suggested and taught by analogy. The teaching thus is made to depend much upon the imagination of the reader, a faculty which in children is illimitable and often fantastic. A little time ago one of the monthly magazines indulged in a symposium giving the opinions of many well-known people as to the propriety of young girls having access to the tree of knowledge, and while some seemed to think that all such teaching was abominable, others opined that judging from the novels that girls read they must know far more about such matters even than their mothers. Perhaps the truth was best expressed by those who said that, if a girl read her Bible and her Shakespeare with understanding, she would know enough. Of course the objection will be raised that in reading these books she must either ask questions or leave much to the imagination. But then the same is true of "Baby Buds." Nevertheless, if a book of this sort is thought desirable, in "Baby Buds" the story will be found both delicately and well told, so far, at least, as it is told at all. We may, however, express our surprise at the suggestion that all this instruction should be given to a child four years old!

Dairy Milk: Its Dangers and the Remedies. By Charles Henry Leet, F.R.C.S. (Liverpool: Tinting and Co. Price 2d.)

Mr. Leet has collected various opinions showing the dangers to infant life caused by the milk trade as now carried on. The various methods of sterilising milk are mentioned, and an apparatus devised by the author for the rapid cooling of milk after domestic sterilisation is described. This little device may, we think, often be found of service not merely for its proper purpose, but for keeping milk cool in hot weather.

### MINOR PUBLICATIONS.

A new illustrated monthly magazine, "Vectis," edited and conducted by Dr. Dabbs, of Shanklin, has just been added to the ranks of journalistic ventures. The number before us for review is interesting and is well got up.

Messrs. Marcus Ward have just issued an illustrated quarterly, price 3d., which is devoted to art and literature as applied to stationery, bookselling, printing, and allied industries. The first issue is both attractive and useful. Art competitions, also, are offered to subscribers.

### BOOKS RECEIVED.

Sanitary Publishing Company.

"Notes on the Factory and Workshop Acts, 1878, 1891, 1895." By D. S. Davies, M.D., and T. H. Gabbicom, A.M.I.C.E.

From the Medical Record, November 20th, 1895.

"Sleep in its Relations to Diseases of the Skin." By L. Duncan Bulkley, A.M., M.D.

### For Reading to the Sick.

### THANKFULNESS.

**Motto.**

Be thankful for what God has done, and He will do more.—*Newton.*

**Verses.**

God gave much peace on earth—much holy joy;
Open'd fountains of perennial spring, whence flow'd
Abundant happiness to all who wish'd
To drink. Not perfect bliss; that dwells with us,
Beneath the eyelids of the Eternal One,
And sits at his right hand alone—but such
As well deserved the name—abundant joy;
Pleasures on which the memories of saints
Of highest glory still delights to dwell.

—*Pollok's "Course of Time."*

Wiser it were to welcome, and make ours,
Whate'er of good; though small the Present brings;
Kind greetings, sunshine, song of birds and flowers,
With a child's pure delight in little things;
And of the griefs unborn to rest secure,
Knowing that mercy ever will endure.

—*R. C. Trench.*

Happy souls! their praises flow
Even in this vale of woe;
Waters in the desert rise,
Manna feeds them from the skies;
On they go from strength to strength,
Till they reach Thy throne at length,
At Thy feet adoring fall,
Who hath led them safe through all.

To me fair memories belong
Of scenes that erst did bless,
Yet no regret, but present song
And lasting thankfulness:—
And very soon to break away,—
Like types,—in fairer things than they.

—*John Keble.*

**Reading.**

Alas! unthankfulness is a frightful and a fatal sign. When the great Apostle, in the first chapter of his Epistle to the Romans, is describing the Gentile world as sunk in ungodliness and idolatry, and traces back their guilt to its source, proving them to be without excuse, because they knew God they glorified Him not as God, he adds, "Neither were thankful." True, therefore, it is, that unthankfulness and ungodliness are usually found together.—*Heavenly Thoughts.*

Christian cheerfulness opens, like spring, all the blossoms of the inmost soul. Try for a single day to preserve yourself in an easy and cheerful frame of mind. Compare the day in which, by thoughts of God, you have rooted out the weeds of dissatisfaction with that on which you have allowed them to grow up, and you will find your heart opened to every good motive, your life strengthened, and your bosom armed with a panoply against every trial of faith. Truly you will wonder at your own improvement.—*Thoughts of Many Minds.*

Just as there is meanness in constant murmuring, so there is a gracefulness and majesty in habitual gratitude; and it is pleasant. It is not the full purse, nor the easy calling, but the full heart, the praising disposition, which makes the blessed life; and of all personal gifts, that man has got the best who has received the quick-discerning eye, the promptly-joyful soul, the ever-praising spirit."—*Mount of Olives.*

It is a low and easy thing to be content; it is too cheap a return for our enjoyments. It concerns us to be highly thankful;—the good Lord make us so.—*Life of Mrs. E. Walker.*

## Figures and Tables

**Figure f1:**